# Research on the construction of the evaluation index system of game-based teaching

**DOI:** 10.3389/fpsyg.2023.1177160

**Published:** 2023-05-23

**Authors:** Yunhong Wang, Sujing Zhang, Hetiao Hong

**Affiliations:** Jing Hengyi School of Education, Hangzhou Normal University, Hangzhou, China

**Keywords:** game-based teaching, class, evaluation index system, literacy cultivation, ANP

## Abstract

This research aims to combine the pursuit of literacy cultivation with a focused investigation into the evidence of game-based teaching (GBT). To achieve this, the study employs a mixed-methods approach including the interview method, Delphi method, and network hierarchical analysis (ANP) to analysis Expert opinions and construct a comprehensive GBT evaluation index system. The results indicated that a comprehensive GBT evaluation index system is comprised of five primary indicators: teaching objectives, game-based teaching methods, teaching content, game-based teaching processes, and game-based teaching characteristics. Additionally, there are 19 secondary indicators, such as objective content, game presentation, context construction, and flow experience. This study expects to effectively capture the unique attributes of game-based classes and to assist teachers in improving the design of GBT activities in practical applications.

## 1. Introduction

Game-based teaching (GBT) has emerged as a cutting-edge and innovative pedagogical approach, garnering significant attention in education policies and reports for its numerous advantages. The World Economic Forum’s report “Schools of the Future: Defining a New Education Model for the Fourth Industrial Revolution,” published in January 2020, highlights the potential impact of game-based teaching and learning on personalized and self-paced learning, and the transformative role it can play in the education system (World Economic Forum, 2020). While GBT has predominantly been implemented in K12 education (Hwang et al., 2016; Müller et al., 2018; Yukselturk et al., 2018), research indicates that it can effectively enhance the learning interests of learners across various age groups, including early childhood education (Gallegos et al., 2017; Tobar-Muñoz et al., 2017; Sung and Hwang, 2018; Wu, 2018; Yu et al., 2018). Evidence suggests that GBT stimulates intrinsic motivation and leads to a significant improvement in learning outcomes. Several studies have demonstrated that the adoption of game-based teaching methods in math and science has resulted in a marked improvement in students’ academic performance (Kim and Ke, 2017; Alzubi et al., 2018; Kiili et al., 2018; Brezovszky et al., 2019). In recent years, game-based teaching has been extending to higher education, displaying a broad range of potential applications (Iosup and Epema, 2014; Chen et al., 2018; Perini et al., 2018).

As GBT gains in popularity, a concerning issue has come to light: well-crafted game-based instruction developed by teachers is often met with reluctance or not validated by experts. As a result, it is imperative to establish an effective game-based teaching evaluation index system that can clarify the fundamental tenets of GBT.

## 2. Literature review

GBT falls under the umbrella of GBL research, which focuses on educational games and game-based learning. The former centers around the existence of independent games, while the latter emphasizes the integration of game-based elements into regular instruction. Both areas share several dimensions, including the evaluation of the approach. The evaluation is challenging in most cases but especially when educational games are used (Serrano et al., 2012). The academic community has conducted numerous evaluation studies on educational games, Petri et al. (2016) improved the model based on MEEGA (a model for assessing the quality of educational games) to assess the perceived quality of educational games from the perspective of player experience and perceived learning. Torres-Toukoumidis et al. (2019) integrated theoretical model of gamification (E-MIGA) translated into an evaluation and quantitative assessment instrument based on expert opinion, which is based on the characteristics of the student population, the teacher role RP, the student role (RA), and other actors in the process (OA) 4 dimensions to evaluate 10 educational games with high download numbers in AppStore (IOS) and PlayStore (Android).some of which are based on the fundamental paradigm of “education+game+x,” such as educational, playful, and technical (Mohamed and Jaafar, 2010; Savi et al., 2011). Ye et al. (2009) has differentiated educational evaluation into cognitive and cognitive process dimensions and incorporated game attributes to establish the evaluation index. Faizan et al. (2019) created a strategy for assessing simulation games used in the classroom, indicating the assessment instruments used at different stages and what to assess in terms of pre-, mid-, and post-game. But as Seaborn says, despite the appearance of forming an increasingly cohesive whole, not all examples of gameplay outside of games can be subsumed under these headings or along these research paths (referring to serious games; Seaborn and Fels, 2015).

Simultaneously, Sailer and Homner (2020) elucidated, through the outcomes of a comprehensive meta-analysis, that gamification in contemporary empirical research constitutes an efficacious pedagogical approach. Game-based teaching (GBT) investigations have predominantly concentrated on academic performance, engagement within a system, and the alacrity of task and assignment execution (Majuri et al., 2018). Examining GBT research from an evaluative standpoint, Tsai et al. (2015) devised an online formative assessment game incorporating multi-objective gaming strategies to probe its effectiveness within an online energy education course. Conversely, Tirado-Olivares (Torres-Toukoumidis et al., 2019) undertook a pre-experimental investigation, employing a mixed-methods modality, utilizing experience points, and a technologically adapted traditional class diary to gage the potential of a gamified formative assessment system. This approach proved effective for assessing learning accomplishments and cross-curricular educational aspects, such as collaborative efforts, classroom interest, and daily task revision. ANA (2019) conceived a game, entitled Downtown, explicitly tailored for individuals with intellectual disabilities, such as Down syndrome, specific Autism Spectrum Disorder (ASD) manifestations, or mild cognitive impairments. The game’s objective is to instruct students in public subway system navigation throughout the city, thereby fostering autonomy and enhancing independent living skills. ANA amassed data on total game session durations, average route completion times, inactivity periods, overall minigame performance, and additional observable factors to establish a distinct construct for validating Downtown’s design and development procedures. A substantial body of GBT assessment research exists, with extant inquiries emphasizing the potential benefits for educators and pupils within classroom settings and how such effects transpire. However, these studies often neglect to address the core concept of GBT, displaying minimal cohesion regarding its theoretical foundations and the essence of gamification (Seaborn and Fels, 2015). A salient inquiry arises: can these impacts be attributed to the diverse ways in which games are incorporated into teaching and learning practices within GBT? In other words, does research bias persist due to inadequate comprehension of GBT characteristics? Alternatively, an excessive focus on individual influences within GBT activities may hinder a comprehensive understanding of the approach. To tackle these concerns, the present study establishes a GBT evaluation index system encompassing the entirety of instructional activities, with educators and students as the principal subjects. The research initially formulates an indicator framework, drawing from an extensive literature review and a voluminous dataset of interview data. Subsequently, the Analytic Network Process (ANP) method is employed to allocate weights to the indicators, while regression analysis is performed to corroborate the framework’s effectiveness. This paper aims to answer the following research questions:

RQ1: What indicators can be utilized to illustrate GBT?

RQ2: Which indicators have the most significant influence on the development of GBT?

RQ3: How can a class utilizing GBT be evaluated?

## 3. Creation of the evaluation index system of GBT

To address the question “What indicators can be used to illustrate GBT?,” the study utilized mixed methods to construct the GBT evaluation index system in three stages. Firstly, the evaluation indices for GBT were developed through the use of the literature research method and interview technique. Secondly, two rounds of expert consultation were conducted *via* the Delphi method to refine the initial indices. Finally, the network hierarchical analysis (ANP) was used to determine the weightage of each indicator, in order to answer the query, “Which indicators have the most significant influence on the development of GBT?”

### 3.1. Theoretical analysis

The system of evaluation indicators for GBT must incorporate the notion that effective teaching can be enjoyable and engaging. The evaluation indices for GBT are centered around two key dimensions: the pedagogical dimension and the game dimension. In terms of pedagogy, both game-based and traditional teaching share the common goal of delivering the curriculum content within a predetermined time and location. Therefore, the study sought to investigate the similarities and differences between the two methods in terms of the pedagogical dimension. Regarding games, the study delved into which aspects of teaching and learning are affected when games (or game mechanisms) are introduced into the classroom. The exploration of this topic was conducted from a theoretical perspective.

#### 3.1.1. Pedagogical dimension

GBT represents an innovation in teaching methods, as it deviates from conventional teaching by incorporating games or game mechanisms. This implies that GBT should be similar to traditional teaching in terms of content. However, GBT offers undeniable advantages in terms of methodology and implementation. In practice, the evaluation of conventional teaching typically revolves around the components of teaching and learning. Table 1 offers a comprehensive overview of the various instruction components.

**Table 1:** The key of instruction.

Reference	The key of instruction
Merrill (2001)	Knowledge components、Instructional strategy components
Tanner (2010)	ENGAGEMENT、EXPLORATION、EXPLANATION、ELABORATION、EVALUATION
Gagné (1986)	motivation, objective, attention, stimulation, learning guidance, retention, transfer, feedback
Aulls and Ibrahim (2012)	teacher roles, student roles, teacher defectiveness, physical setting, social environment, emotional environment, activities, time allocation, material, discourse, content.
Dick et al. (2005)	teacher, learner, instructional materials, and learning environment.
Slavin (1995)	Quality of Instruction、Appropriate Levels of Instruction、Incentive、Time
Algozzine and Ysseldyke (1992)	Instruction content、objective、preparing for instruction、time、environment、motivating students、feedback、practice、decisions、judgments
Levin and Long (1981)	Educational Objectives、Questions (utilized by teachers)、Visual Aids、Practice
Van Merriënboer et al. (2002)	Learning Tasks、Supportive Information、Just-in-Time Information、Part-task Practice

Table 1 highlights that evaluation of instruction is often closely linked to components such as teachers, students, content, and methods. These categories are fundamental aspects that must be considered when constructing an instruction evaluation index system. Teachers and students are crucial components of the instruction relationship, but they cannot be directly evaluated. Therefore, they are typically incorporated into the evaluation indices as a specific perspective in the development of the evaluation index system. Consequently, this study focuses on six main indicators for the evaluation index system: objectives, content, process, methods, environment, and media.

#### 3.1.2. Game dimension

The incorporation of games (or game mechanics) in teaching and learning is the defining factor that sets it apart from conventional teaching methods. According to Kapp’s definition (Kapp, 2012), games are comprised systems, players, abstraction, challenge, rules, interactivity, feedback, quantifiable outcomes, and emotional responses, and these factors combine to revolutionize teaching and learning. In games, “abstraction” refers to the representation of reality within a virtual context that retains some aspects of reality. In teaching, this concept is often referred to as “contextualization.” The presence and necessity of contextualization in both games and teaching confirms its validity as a category in the evaluation of GBT. Feedback mechanisms play a crucial role in promoting greater immersion in play (Law and Chen, 2016; Pilegard and Mayer, 2016; Yang, 2017). Thus, the timeliness of feedback needs to be explicitly addressed in the evaluation indices. Some research indicates that autonomy, competence, and relevance independently predict enjoyment and future engagement in play (Ryan et al., 2006). In other words, the enjoyment of play emphasizes the degree of autonomy, competence, and relevance of the play to the content in the gamification process, which serves as a precondition for assessing whether the play is fun or not. This is a prerequisite for evaluating whether the game is enjoyable or not. Therefore, the evaluation items of timeliness of feedback, the comprehensiveness of the process, and contextualization have been extracted from the game dimension.

### 3.2. Constructing indicators based on grounded theory

Based on the previous discussion of the two dimensions of teaching and play, the researchers have identified primary indicators such as objectives, content, process, methods, environment, and media, as well as secondary evaluation indicators such as timeliness of feedback, process fun, and contextuality. However, to refine the main indicators into operational and specific indicators, and to take into account any characteristics of GBT that still need to be considered, the researchers conducted in-depth interviews with over 20 teachers who use GBT in practice. The original interview data underwent an open coding process, where the researchers aimed to restore the teachers’ evaluation concept of GBT as exhaustively and comprehensively as possible by retaining the teachers’ original words and text while focusing on measurable relevant statements and concepts. Table 2 illustrates the open coding process for one teacher’s interview data.

**Table 2:** Open coding table for some sample data.

Interview data material	Open coding
It (meaning to conduct game-based teaching) requires both game-based thinking and, for example, contextualized thinking	Game-based teaching needs context
It’s no longer a very straightforward way of being taught by an elementary teacher. I can transform it into a game idea, or challenging or fun activity, or a way of playing a game, just transforming it into a form. It’s like many of these online games, online games, board games, it’s actually in this form, but it’s not necessarily in the software form like this undeniable game in Shenzhen. It can be a game of life.	To construct play activities that are different from the regular teaching
(Connected to life) I should say that it is already present in many classes now, and it is practically necessary to teach class life scenarios	Play-based teaching needs to be linked to life
The teacher has to create a situation which may sometimes be real, sometimes a virtual one, and sometimes an interplay of the virtual and the real.	Teachers need to create context in the class
······	

The researchers proceeded with an Axial Coding analysis of all the open coding extracted, which involved summarizing and grouping codes with similar essential attributes. For instance, codes such as “objectives should be scientific and reasonable,” “objectives should be specific,” and “objectives should be hierarchical” were coded as “scientific.” The code “teachers should create situations in the class” was coded as “contextual.” This process resulted in 19 Axial codings. Subsequently, the researchers carried out a selective coding process that combined the instructional elements and extracted three core categories: the teacher category, the student category, and the classroom game interaction category. The process of selective coding is presented in Table 3.

**Table 3:** Selective coding.

Category	Associated categories	Concept analysis
Teachers	Instructional objective (Schoenfeld-Tacher and Sims, 2013)	Scientific
		Clarity
	Instructional methods (Tay et al., 2022)	playfulness
Students	Emotional constructs (Loderer et al., 2020)	High-level thinking
		Emotional regulation
		Subject-active
		Games for fun
	Learning styles (Hwang et al., 2012)	Ability to cooperate
		Sharing philosophy
Game interaction	Instructional contents (Gunter et al., 2007)	Openness
		Practicality
		Contextuality
	Instructional process (Ucus, 2015)	Suitability of activities
		Timeliness of feedback
		Process fun
		Disciplinary stability
	Instructional atmosphere (Wang, 2015)	Interactivity
		Cooperative
		Democracy

After processing the interview data, the study created a preliminary GBT evaluation index system that uses the selective codings as the main categories and associated categories as the primary indicators. The system consists of eight primary indicators, including teaching objectives, instructional methods, and emotional constructs, as well as 20 secondary indicators, such as scientificity, clarity, and playfulness. The primary indicators represent the essential aspects of GBT, while the secondary indicators provide a more detailed and specific evaluation of these aspects. The system aims to provide a comprehensive and objective evaluation of GBT that can help teachers design and implement effective GBT activities.

### 3.3. Delphi-based indicator revision

To ensure the scientific validity of the evaluation indicators and eliminate the influence of theoretical constructs and subjective elements from the interview process, the study used the Delphi method to revise the initially constructed evaluation indicators. The researcher selected 10 experts in the field of game-based learning, including three professors, two associate professors, and five teachers who use GBT, including two expert teachers. In the first round of specialist consultation, the experts pointed out several problems with the proposed index system. These included the slightly confusing dimensions of the indicators at the same level, the lack of uniformity in textual descriptions, and some indicators failing to reflect the characteristics of game-based teaching. As an example, Table 4 shows the statistical results of some secondary indicators and their revision opinions.

**Table 4:** Statistical results of secondary indicators and modifications.

Secondary indicators	Mean	SD	Results
GB1 Scientific	9.29	0.983	Amend to read “Objective content”
GB2 Clarity	9.00	0.816	Amend to “Architecture”
Added: “Value Objectives”，Merged from the original GA4 learning approach			
Added: “Competence Objectives”，Merged from the original GA3 emotional construct			
GB3 playfulness	7	2.582	Amend to “GB5 Game Presentation”
GB4 Indirectness	6.43	2.225	Amend to “GB6 expression pathway”
GB5High-level thinking	7.714	1.704	delete
......			

The second round of expert consultation received significantly fewer opinions, but some experts still proposed revisions to the indicators. Eventually, one secondary indicator was deleted, and the detailed descriptions of two indicators were revised. The mean, standard deviation, and coefficient of variation of experts’ scores in the second round of consultation were significantly better than those in the first round, indicating that the experts had reached a consensus. As a result, the “GBT Evaluation Index” was revised based on two rounds of expert consultation, which consists of 5 primary indicators and 19 secondary indicators. These are listed in Table 5.

**Table 5:** Evaluation index of GBT.

Primary indicators	Secondary indicators
Instructional objectives	System architecture
	Objectives
	Value objectives
	Competence objectives
Instructional contents	Knowledge system
	Contextual constructs
	Difficulty gradient
	Target oriented
Game-based teaching process	Game content
	Immediate feedback
	Multidimensional emotions
	Games as subjects
	Flow experience
Game-based teaching methods	Game presentation
	Means of expression
	Interactive orientation
Game-based teaching features	Game roles
	Class atmosphere
	Teaching rhythm

### 3.4. Weighting

The indicator entries describe factors to be considered in teaching and learning, but they do not indicate the importance of each factor in influencing teaching and learning activities. This study used a weighting system to determine the relative importance of each factor. The network hierarchical analysis (ANP) was chosen to assign weights as it is specific to the field of education and teaching, where many indicators interact and provide feedback dynamically in a classroom setting.

**Figure 1:**
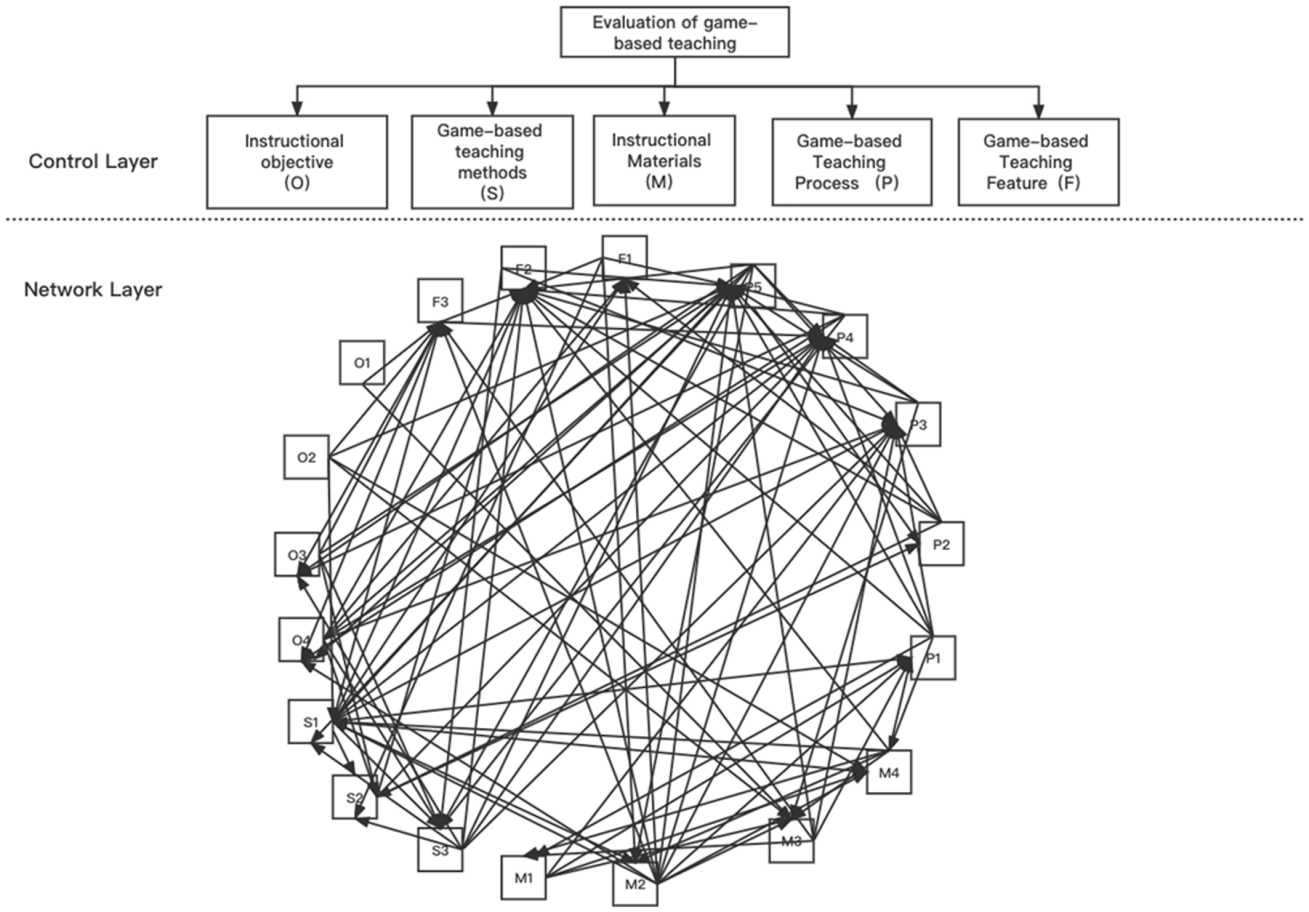
The network structure of the evaluation index system for GBT.

The researchers established the network structure of the index system, with “Evaluation of GBT” as the main objective layer, and five primary indicators as the control criterion layer, including instructional objectives (O), game-based teaching methods (S), instructional materials (M), game-based teaching processes (P), and game-based teaching feature (F). The 19 secondary indicators were divided into different sets of components and numbered as O1 (system architecture), O_2_ (objective), M1 (knowledge system), and M2 (contextual construction), respectively. Based on the above settings, the study established the network structure of the GBT evaluation system, as shown in Figure 1.

To obtain the weights of the secondary indicators, the researchers followed the same procedure as with the primary indicators, using ANP to establish a multi-level network structure of the evaluation system. The researchers then issued another ANP Expert Opinion Questionnaire to the same group of five experts, asking them to judge the relative importance of the secondary indicators within their corresponding primary indicator sets. The experts were asked to provide a judgment matrix for each set of secondary indicators, and the weights were calculated using the ANP method. The final weights of the secondary indicators were obtained by multiplying the weight of the corresponding primary indicator by the weight of the corresponding secondary indicator within the set. The weights of the secondary indicators are included in the complete evaluation index system presented in (Table 6).

**Table 6:** The weighting of the primary indicators of the evaluation system of GBT.

	Instructional objectives	Instructional materials	Game-based teaching processes	Game-based teaching methods	Game-based teaching features
Instructional objectives	0.27729	0.27729	0.27729	0.27729	0.27729
Instructional materials	0.28628	0.28628	0.28628	0.28628	0.28628
Game-based teaching processes	0.15883	0.15883	0.15883	0.15883	0.15883
Game-based teaching methods	0.20765	0.20765	0.20765	0.20765	0.20765
Game-based teaching features	0.06994	0.06994	0.06994	0.06994	0.06994

After multiple rounds of expert consultation, data coding, and statistical analysis, this study has established a comprehensive evaluation indicator system for GBT that considers the pedagogical dimension and the game dimension, as shown in Table 7. The evaluation system consists of five primary indicators (Instructional objectives, game-based teaching methods, instructional materials, game-based teaching processes, and game-based teaching features) and 19 secondary indicators. The weightings of each indicator have been calculated using the ANP method and are included in the evaluation system. This GBT evaluation system can provide guidance for teachers and educators to create effective and engaging game-based learning environments that foster conceptual understanding and personal development.

**Table 7:** The evaluation index system of GBT (with weights).

Primary indicators	Secondary indicators	Detailed description
Instructional objectives (0.27729)	System architecture 0.05972 (0.00886*)	A hierarchical system of instructional objectives, with sub-objectives that fully cover the requirements of the primary objectives
		Objectives 0.05972 (0.00886*)	The instructional objectives are clearly described, specific, expressed as concrete behavioral objectives and easy to achieve
	Value objectives 0.48484 (0.07193*)	The instructional objectives focus on the development of the concept of sharing and cooperation among students
	Competence objectives 0.39573 (0.05871*)	The instructional objectives emphasize the development of students’ higher-level thinking skills, such as decision-making and problem-solving skills.
	Instructional Materials (0.28628)	Knowledge system 0.22893 (0.04616*)	Accurate teaching materials with no scientific errors
Contextual constructs 0.29381 (0.05925*)		game-based instructional materials is contextual, abstracting game situations from real life
Difficulty gradient 0.24939 (0.05029*)		The instructional materials is able to highlight important and difficult points and capture key
Target oriented 0.22787 (0.04594*)		The instructional materials and the content of the game reflect the instructional objectives, through the content of the game, the predetermined teaching objectives can be observed
game-based teaching processes (0.15883)	Game content 0.11520 (0.03321*)	The game activities (or game mechanics) that occur in game-based class are age-appropriate and applicable to the classroom and are easily accessible to students of the current school level
	Immediate feedback 0.15197 (0.04381*)	timely teacher (or technology) feedback during game-based teaching
	Multidimensional emotions 0.19661(0.05668*)	game-based teaching in which teachers and students have fun with playful activities and playful emotions such as fun, enjoyment or tension
	Games as subjects 0.13959 (0.04024*)	Students are the main focus of play activities in game-based teaching, and the frequency of student speech and action is slightly greater than that of teacher speech and action
	Flow experience 0.39663 (0.11435*)	High levels of student engagement in game-based teaching, with the vast majority of students immersed in the gamification process
game-based teaching methods (0.20765)	Game presentation 0.27800 (0.06943*)	A game-based teaching method requires the use of games (or game mechanics) as the main (or supporting) tool, either as a game for the whole lesson or as a gamified minimum unit of “core play + game elements”
	Means of expression 0.25309 (0.06322*)	Game-based teaching methods require more indirect teaching methods to help students construct knowledge, such as asking questions, encouraging, guiding, etc.
	Interactive orientation 0.46891 (0.11711*)	Game-based teaching methods should place more emphasis on cooperation and competition between students
game-based teaching features (0.06994)	Game roles 0.05670(0.00634*)	There is a certain shift in the role of the teacher to that of a ‘gamer’, a ‘spectator’”
	Class atmosphere 0.74762 (0.08367*)	Effective division of labor between teachers and students in game activities, showing an atmosphere of equality and harmony
	Teaching rhythm 0.19568 (0.02190*)	The games (or elements of games) used in the game-based class allow for a relaxed, rhythmic and relaxed classroom.

*Indicates global weights.

## 4. Validation of the evaluation index system of GBT

An experiment was conducted to answer the second research question, and the results are reported in subsequent sections.

### 4.1. Experimental procedure

Two undergraduate students majoring in mathematics and interested in GBL were invited to evaluate five game-based class video recordings using the GBT evaluation index system developed in this study. The two raters were selected based on their background and experience in education and GBL. They were both teacher trainees and had some teaching experience, and their research interests were also related to GBL. The raters evaluated the recordings using the GBT evaluation index system and provided feedback on the clarity and usefulness of the system. The feedback was used to further refine the evaluation index system.

In the first stage of the study, the inter-rater agreement between the two raters was tested for reliability using the evaluation index system of GBT. Given that there were only two raters in this study, Spearman’s rank correlation was used to analyze their reliability. The results showed that the reliability coefficient of the two raters reached 0.9 (Sig = 0.037 < 0.05), indicating a high level of agreement between the two observers when using this index system for evaluation.

The dependent variable in the regression analysis is the total score of the scale used in this study. Before conducting the analysis, the normality of the data was tested using both the normality test (Sig1 = 0.106 > 0.05; Sig2 = 0.32 > 0.05) and the Chi-square test (Sig = 0.551 > 0.05), which indicated that the hypothesis of normal distribution of the data cannot be rejected, and the homogeneity of variance can be assumed.

In the second stage of the study, the validity of each of the five primary indicators was verified by subjecting their scores to a linear regression analysis with the overall scale scores. This analysis aimed to use the scores of each primary indicator to predict the scores of the overall scale and determine their validity. A high coefficient of determination (*R*^2^) would indicate a strong correlation between the scores of the primary indicators and the overall scale.

### 4.2. Results

#### 4.2.1. “Instructional objectives” score as a predictor

**Figure 2:**
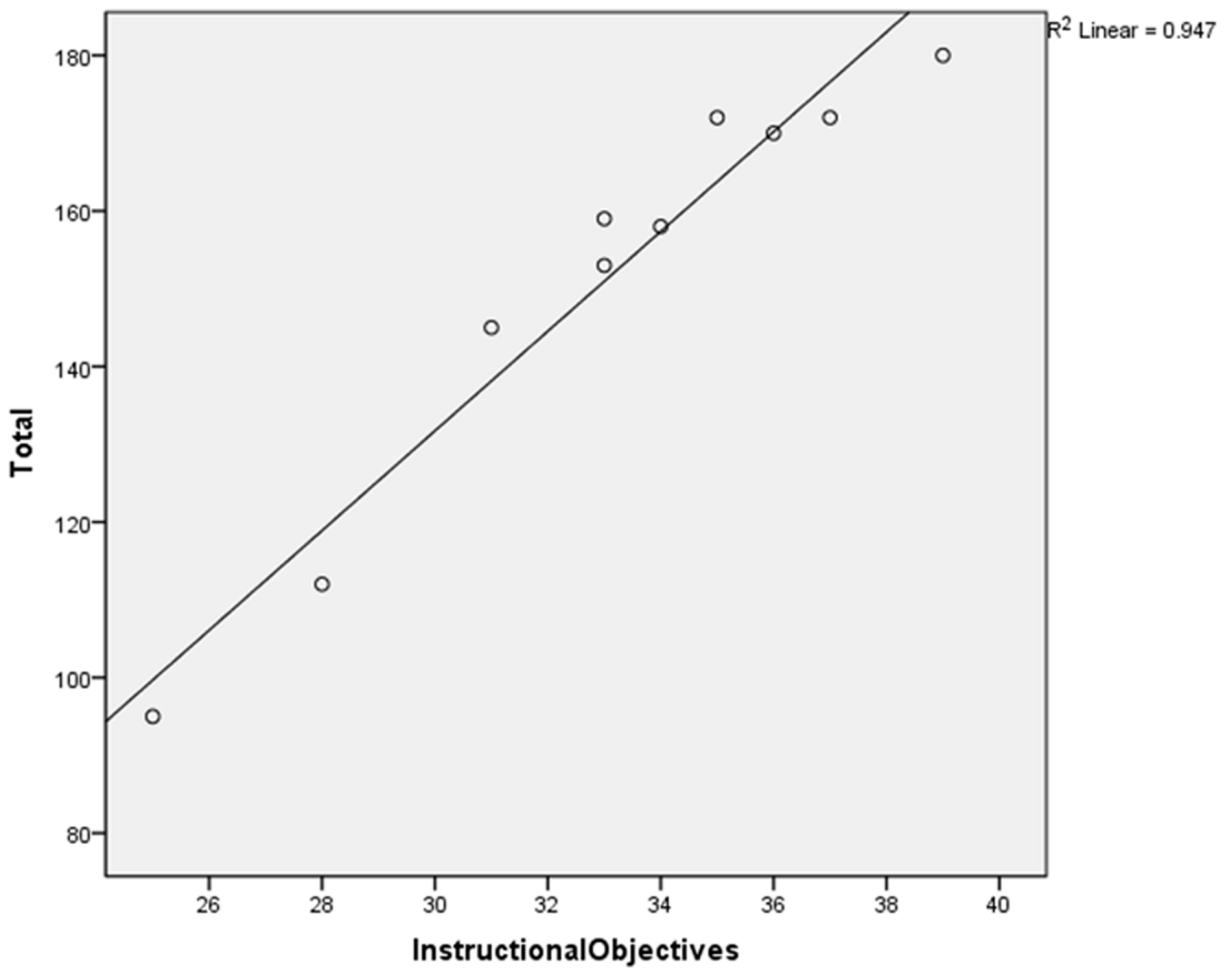
The prediction plot.

After conducting the regression analysis, a high coefficient of determination (*R*^2^) was found when using “Instructional objectives” to predict the total score (*R*^2^ = 0.947, Sig = 0.000 < 0.05), as shown in Table 8. Figure 2 displays the scatter plot and fit line of the “Instructional objectives” score used to predict the overall scale score.

**Table 8:** Regression model of instructional objectives.

Model	*R*	*R*^2^	Adjusted *R*^2^	Std. Error of the Estimate	*R*^2^ Change	*F* Change	Df1	Df2	Sig. F Change	Durbin-Watson
1	0.973	0.947	0.941	6.740	0.947	143.683	1	8	0.000	3.2777

The study proceeded to conduct an ANOVA test on the regression relationship to determine the presence of a significant linear regression relationship between the overall scale scores and the “Instructional objectives” indicator scores. The results of the regression ANOVA demonstrated a significant linear regression between the “Instructional objectives” indicator scores and the overall scale scores (*F* = 143.683, *p* = 0.000 < 0.001). Hence, it can be inferred that the indicators of the “Instructional objectives” dimension can effectively predict the overall scale scores.

#### 4.2.2. “Game-based teaching methods” score as a predictor

**Figure 3:**
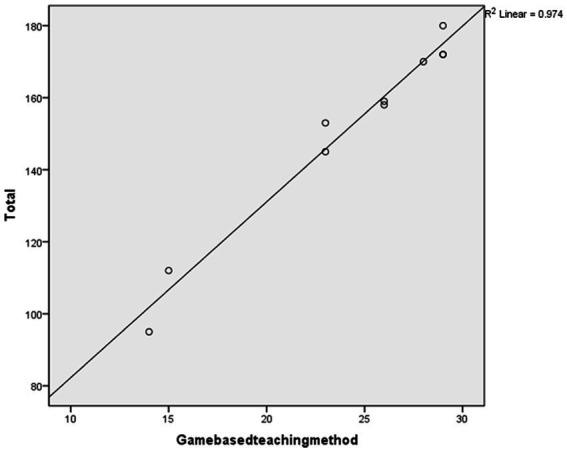
The prediction plot.

After conducting the regression analysis, a high coefficient of determination (*R*^2^) was found when using “game-based teaching methods” to predict the total score (*R*^2^ = 0.974, Sig = 0.000 < 0.05), as shown in Table 9. Figure 3 displays the scatter plot and fit line of the “game-based teaching methods” score used to predict the overall scale score.

**Table 9:** Regression model of game-based teaching methods.

Model	*R*	*R*^2^	Adjusted R^2^	Std. Error of the Estimate	*R*^2^ Change	*F* Change	Df1	Df2	Sig. F Change	Durbin-Watson
1	0.987	0.974	0.971	4.723	0.974	300.838	1	8	0.000	1.465

The study proceeded to conduct an ANOVA test on the regression relationship to determine the presence of a significant linear regression relationship between the overall scale scores and the “game-based teaching methods” indicator scores. The results of the regression ANOVA demonstrated a significant linear regression between the “game-based teaching methods” indicator scores and the overall scale scores (*F* = 300.838, *p* = 0.000 < 0.001). Hence, it can be inferred that the indicators of the “game-based teaching methods” dimension can effectively predict the overall scale scores.

#### 4.2.3. “Instructional materials” score as a predictor

**Figure 4:**
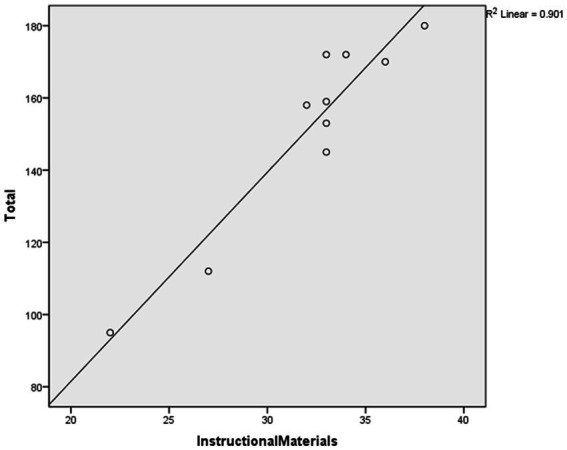
The prediction plot.

After conducting the regression analysis, a high coefficient of determination (*R*^2^) was found when using “Instructional Materials” to predict the total score (*R*^2^ = 0.901, Sig = 0.000 < 0.05), as shown in Table 10. Figure 4 displays the scatter plot and fit line of the “Instructional Materials” score used to predict the overall scale score.

**Table 10:** Regression model of instructional material.

Model	*R*	*R*^2^	Adjusted *R*^2^	Std. Error of the Estimate	*R*^2^ Change	*F* Change	Df1	Df2	Sig. F Change	Durbin-Watson
1	0.949	0.901	0.889	9.234	0.901	72.803	1	8	0.000	1.422

The study proceeded to conduct an ANOVA test on the regression relationship to determine the presence of a significant linear regression relationship between the overall scale scores and the “Instructional Materials” indicator scores. The results of the regression ANOVA demonstrated a significant linear regression between the “Instructional Materials” indicator scores and the overall scale scores (*F* = 72.803, *p* = 0.000 < 0.001). Hence, it can be inferred that the indicators of the “Instructional Materials” dimension can effectively predict the overall scale scores.

#### 4.2.4. “Game-based teaching process” score as a predictor

**Figure 5:**
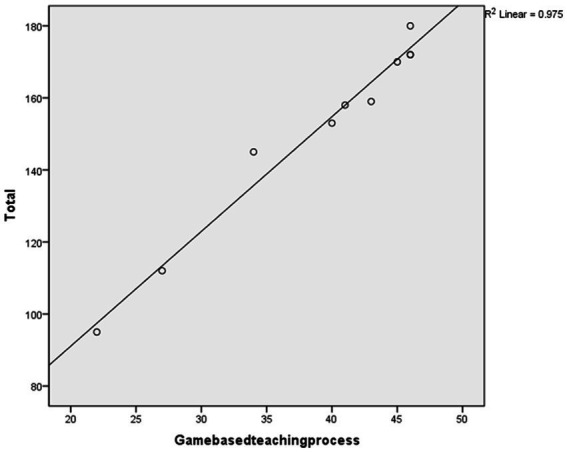
The prediction plot.

After conducting the regression analysis, a high coefficient of determination (*R*^2^) was found when using “game-based teaching process” to predict the total score (*R*^2^ = 0.974, Sig = 0.000 < 0.05), as shown in Table 11. Figure 5 displays the scatter plot and fit line of the “game-based teaching process” score used to predict the overall scale score.

**Table 11:** Regression model of game-based teaching process.

Model	*R*	*R*^2^	Adjusted *R*^2^	Std. Error of the Estimate	*R*^2^ Change	*F* Change	Df1	Df2	Sig. F Change	Durbin-Watson
1	0.987	0.975	0.972	4.631	0.975	313.331	1	8	0.000	1.922

The study proceeded to conduct an ANOVA test on the regression relationship to determine the presence of a significant linear regression relationship between the overall scale scores and the “game-based teaching process” indicator scores. The results of the regression ANOVA demonstrated a significant linear regression between the “game-based teaching process” indicator scores and the overall scale scores (*F* = 313.331, *p* = 0.000 < 0.001). Hence, it can be inferred that the indicators of the “game-based teaching process” dimension can effectively predict the overall scale scores.

#### 4.2.5. “Game-based teaching feature” score as a predictor

**Figure 6:**
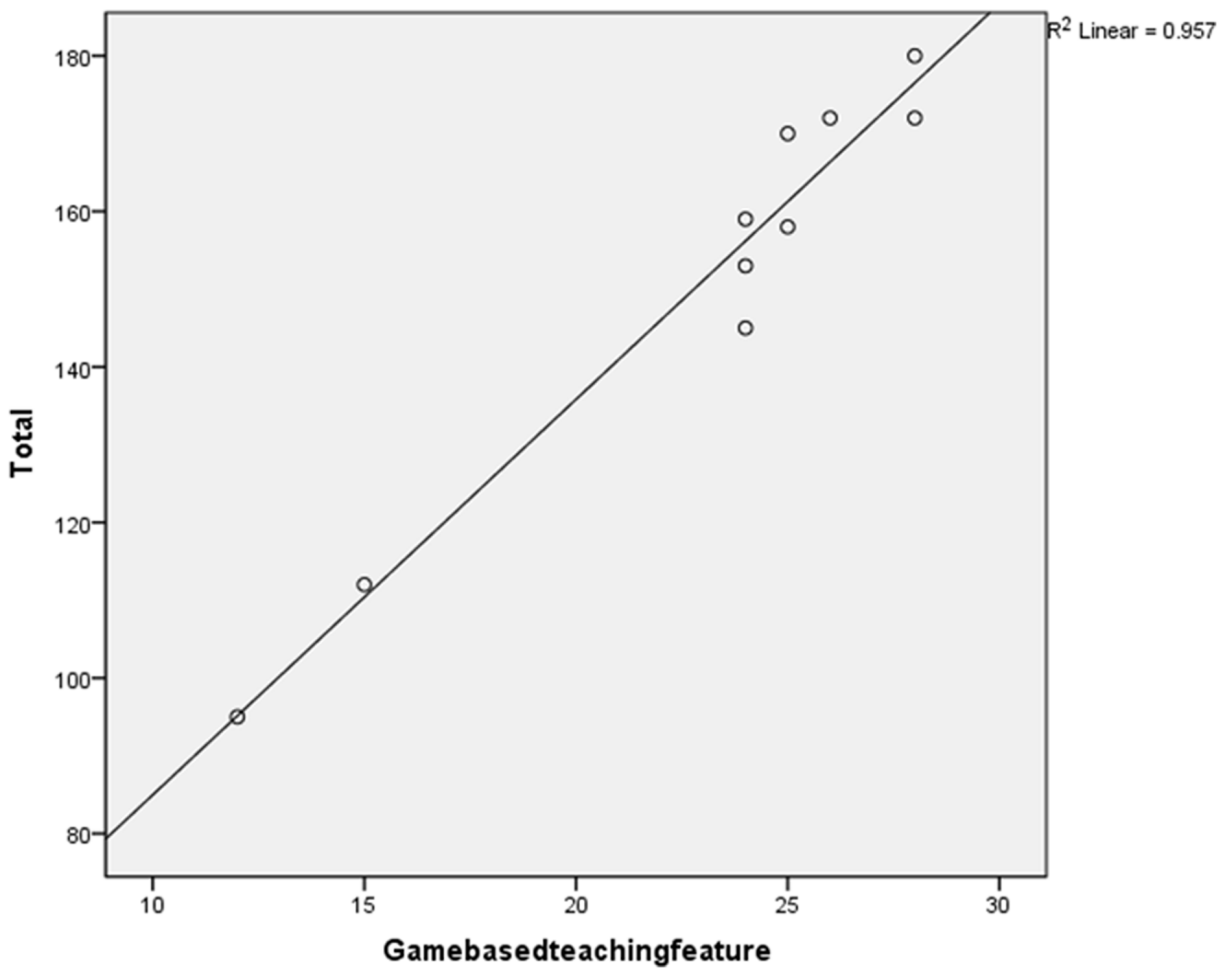
The prediction plot.

After conducting the regression analysis, a high coefficient of determination (*R*^2^) was found when using “game-based teaching feature” to predict the total score (*R*^2^ = 0.957, Sig = 0.000 < 0.05), as shown in Table 12. Figure 6 displays the scatter plot and fit line of the “game-based teaching feature” score used to predict the overall scale score.

**Table 12:** Regression model of game-based teaching feature.

Model	*R*	*R*^2^	Adjusted R^2^	Std. Error of the Estimate	*R*^2^ Change	*F* Change	Df1	Df2	Sig. F Change	Durbin-Watson
1	0.978	0.957	0.952	6.093	0.957	177.588	1	8	0.000	1.830

The study proceeded to conduct an ANOVA test on the regression relationship to determine the presence of a significant linear regression relationship between the overall scale scores and the “game-based teaching feature” indicator scores. The results of the regression ANOVA demonstrated a significant linear regression between the “game-based teaching feature” indicator scores and the overall scale scores (*F* = 177.588, *p* = 0.000 < 0.001). Hence, it can be inferred that the indicators of the “game-based teaching feature” dimension can effectively predict the overall scale scores.

## 5. Discussion

Now, let us return to the research questions of this study:

What indicators can be utilized to illustrate GBT? To achieve this goal, the study first identified two core dimensions of GBT: instructional and game. Based on these dimensions, the study developed a new scale consisting of 5 primary and 29 secondary indicators using a theory-based approach. Upon examining the aforementioned premises, it becomes evident that Game-Based Teaching (GBT) encompasses five distinct categories: instructional objectives, instructional materials, game-based teaching processes, game-based teaching methods, and game-based teaching features. The majority of scholars who have endeavored to delineate the factors integral to devising instructional programs have emphasized the importance of identifying objectives (Glaser, 1966). If educators can effectively devise instructional strategies, anticipated instructional goals should be attainable through predetermined accomplishments, contingent upon student needs (Myers et al., 2017; Browne, 2018; Rapson, 2018). Instructional objectives constitute the cornerstone of GBT, suggesting that when designing GBT for learners, meticulous consideration should be given to which learner needs can be fulfilled by instructional objectives.

Instructional materials pertain to the resources through which learners construct individual knowledge. Moreover, some scholars posit that instructional design (ID) is the scientific discipline concerned with designing, developing, evaluating, and maintaining instructional materials to create comprehensive specifications that facilitate learning and performance (Martin, 2011). Consequently, instructional materials delineate the boundaries of GBT and circumscribe learners’ experiences.

Of the five domains, the most crucial are game-based teaching processes, game-based teaching methods, and game-based teaching features. These domains elucidate the necessity for GBT to actively involve learners in game activities, as GBT not only generates games for learners but also crafts learning activities that progressively introduce concepts and guide users toward the ultimate objective (Pho and Dinscore, 2015). In this context, learners transition from mere consumers to producers of content through their actions and decision-making processes (Gee, 2003). The merit of this study’s indicator system, which portrays the comprehensive framework of GBT through five categories, lies in its capacity to offer a holistic perspective on the influence of play (or gamification) on teaching and learning Activities.

Which indicators have the most significant influence on the development of GBT? In light of the weighted schema employed in the indicator framework, we suggest that Instructional Materials play a crucial role in the progression of Game-Based Teaching (GBT). Some scholars have remarked on the limited presence of games capable of empirically showcasing effectiveness in delivering academic content when used independently in educational contexts (Garris et al., 2017). GBT often receives praise for its propensity to encourage student engagement in gameplay and promote positive social interactions (Gunter et al., 2007). Shi hypothesizes that this occurrence may be attributed to game designers’ skill in devising intriguing games, without necessarily preserving material quality, while educators concentrate on potent instructional resources but may struggle with creating engaging games (Shi and Shih, 2015). This study aligns with the insights of Shi et al. the underlying objective of GBT is not solely to provide amusement and playfulness, but rather to work toward achieving germane academic goals (Cheng and Su, 2012). This leads to the question of how best to design instructional materials that support students’ academic growth. Gunter et al. (2007) contends that integrating newly acquired content within contextual settings is essential for the success of games in reinforcing students’ learning. Fu et al. (2019)highlight the considerable potential of authentic scenarios in making learning more meaningful. The “Contextual Constructs” indicator in the framework, which achieved the highest weight within the Instructional Materials dimension, endorses these viewpoints. This finding suggests that a key consideration in GBT implementation rests in the careful design of Instructional Materials and that ensuring high-quality Contextual Constructs is a vital precondition for realizing GBT.

How can a class utilizing GBT be evaluated? Through the regression analysis, it was discerned that each of the primary indicators more accurately forecasted the cumulative scale scores; consequently, the evaluative framework devised in this investigation succeeded in encouraging disparate evaluators to appraise classrooms employing GBT uniformly, predicated on the content of the indicators. Enhancing the caliber of instruction and learning constitutes a pivotal element in the majority of proposals aimed at augmenting school quality (Hanushek et al., 2016). Certain investigations have advocated the utilization of feedback surveys, inspections (*via* peer observation), and enigmatic clientele (students) as the three principal methods for monitoring service quality in higher education (Douglas and Douglas, 2006). Educators can elevate the quality of their instruction by refining their pedagogical activities (Hau, 1996). As such, evaluating instruction and learning entails assessing instructors (and their classroom interactions). Educator assessment, designed to enhance instructional quality, embodies a formative assessment that molds teachers’ performance to render it more efficacious (Popham, 1988). The indicator system we devised represents a formative evaluation framework that assists educators in acquiring impartial outcomes in the formative assessment of GBT and in receiving comprehensive guidance within such assessments to bolster the quality of instruction and learning.

## 6. Conclusions and future work

This study has developed a GBT evaluation index system with three main considerations. Firstly, it emphasizes the guiding role of the index system and provides an evaluation system for various game-based teaching activities. Secondly, it pays attention to the goal orientation of literacy cultivation to avoid the evaluation indexes leading to a cycle of “literacy without teaching.” Lastly, it focuses on the critical status of sub-disciplinary teaching and meets the established requirements of the index system for sub-disciplinary teaching.

As the indicator system demonstrates, GBT reflects the differences between its and other instruction methods in terms of both instruction and game. We can now provide a powerful response to the basic principles of GBT, namely, effective teaching and fun and games. This principle brings a fresh perspective to GBT, helping us to look at the impact that the vehicle of play brings to teaching and learning and how these impacts occur in the context of the totality of teaching and learning activities. It is worth noting that the assessment function of the indicator system may be an opponent of teachers in some cases, so we need to emphasize that the purpose of the GBT evaluation index system is not to grade teachers who use GBT but to establish a reference system that educators can use to assess their GBT practices. Teachers should utilize this system to reflect on and improve their GBT approaches (Popham, 1988), identify any areas for improvement, and promote the advancement of teaching reform in schools.

This study has focused on evaluating teaching within the field of GBT, but it has not defined the problem and has not conducted an in-depth investigation on classes of different subjects and levels of students. Methodologically, the number of samples for comparison in the difference comparison study is relatively small, which may limit the generalizability of some of the conclusions. In using the Delphi method, the study chose only 10 experts, which may have limited the scope of the evaluation.

Considering the limitations of this study, several suggestions are proposed for future research. Firstly, it is recommended to incorporate students’ views and opinions on GBT in the index system to broaden its scope. Secondly, to ensure the generalizability of the research findings, future studies should increase the sample size of experts, teachers, and GBT recordings. Lastly, it is suggested that different subjects and class levels be included to explore the applicability of the evaluation indicators. These improvements could enhance the comprehensiveness and reliability of the evaluation index system, and provide more practical guidance for GBT practice.

## Data availability statement

The raw data supporting the conclusions of this article will be made available by the authors, without undue reservation.

## Author contributions

YW and SZ: conceptualization. HH: methodology. YW: software, validation, and investigation. SZ: resources, supervision, and funding acquisition. YW and HH: writing–original draft preparation. HW and SZ: writing–review and editing. All authors have read and agreed to the published version of the manuscript.

## Funding

This research work was supported by the “13th Five-Year Plan” Teacher Education Innovation Project of Zhejiang Province: “Digital Competence Development of Future Teachers” Office of Zhejiang Provincial Education Department [2017], No. 80 and 2021 Ministry of Education Industry-University Cooperation Collaborative Education Project: “Construction of Virtual Reality Teaching Practice Base of Hangzhou Normal University” (202102464071).

## Conflict of interest

The authors declare that the research was conducted in the absence of any commercial or financial relationships that could be construed as a potential conflict of interest.

## Publisher’s note

All claims expressed in this article are solely those of the authors and do not necessarily represent those of their affiliated organizations, or those of the publisher, the editors and the reviewers. Any product that may be evaluated in this article, or claim that may be made by its manufacturer, is not guaranteed or endorsed by the publisher.
